# Surgical decision-making for individuals with differences of sex development: Stakeholders’ views

**DOI:** 10.3389/fruro.2023.1092256

**Published:** 2023-02-24

**Authors:** Erica M. Weidler, Melissa Gardner, Kristina I. Suorsa-Johnson, Tara Schafer-Kalkhoff, Meilan M. Rutter, David E. Sandberg, Kathleen van Leeuwen

**Affiliations:** ^1^ Division of Pediatric Surgery, Phoenix Children’s, Phoenix, AZ, United States; ^2^ Accord Alliance, Higley, AZ, United States; ^3^ Susan B. Meister Child Health Evaluation & Research (CHEAR) Center and Division of Pediatric Psychology, Department of Pediatrics, University of Michigan Medical School, Ann Arbor, MI, United States; ^4^ Division of Pediatric Psychology and Behavioral Health, Department of Pediatrics, University of Utah Spencer Fox Eccles School of Medicine, Salt Lake City, UT, United States; ^5^ Division of Endocrinology, Cincinnati Children’s Hospital Medical Center, Cincinnati, OH, United States; ^6^ Department of Pediatrics, University of Cincinnati College of Medicine, Cincinnati, OH, United States

**Keywords:** differences of sex development (DSD), disorders of sex development (DSD), intersex, shared decision-making (SDM), genital surgery, gonadal surgery, decision aids, proxy decision-making

## Abstract

**Introduction:**

Advocacy and human rights organizations have called for a moratorium on elective surgical procedures until the patient is able to fully participate in the decision-making process. Due to the controversial nature surrounding surgery in differences of sex development (DSD) care, we aimed to assess the factors that teens and adults with DSD, parents, healthcare providers and other allied professionals consider pertinent to complex surgical decisions in DSD.

**Methods:**

Stakeholders (n=110) in DSD care participated in semi-structured interviews exploring features and potential determinants of successful healthcare outcomes. Audio-recordings were transcribed, coded, and analyzed using qualitative data software. Codes for “Process of Decision-Making” and “Successful Outcome–Surgery/Appearance/Function” were further searched using keywords “surgery,” “procedure,” and “timing.”

**Results:**

Several themes were identified: 1) The nature or type of the decision being made; 2) Who should be involved in the decision-making process; 3) Timing of conversations about surgery; 4) Barriers to decision-making surrounding surgery; 5) The elements of surgical decision-making; and 6) The optimal approach to surgical decision-making. Many stakeholders believed children and adolescents with DSD should be involved in the process as developmentally appropriate.

**Conclusion:**

DSD include a wide range of diagnoses, some of which may require urogenital reconstruction to relieve obstruction, achieve continence, and/or address other anatomical differences whether cosmetic or functional. Adolescents and adults with DSD desired autonomy and to be part of the decision-making process. Parents were divided in their opinion of who should be involved in making elective surgical decisions: the child or parents as proxy medical decision-makers. Providers and other professionals stressed the importance of process and education around surgical decisions. Ongoing research examines how decision-makers evaluate tradeoffs associated with decision options.

## 1 Introduction

Differences of sex development (DSD)^1^ is an umbrella term covering a wide range of congenital conditions in which development of chromosomal, gonadal or anatomic sex is atypical (1). DSD encompass a wide range of phenotypes and care pathways, not all of which include a surgical component. Surgical interventions in DSD fall in four main categories: 1) Reconstruction of external genital tissue to achieve more typical appearance and function (e.g., genitoplasty, hypospadias repair); 2) Repair related to obstruction or absence of Müllerian structures (e.g., creation of vaginal opening); 3) Gonadal removal to eliminate tumor risk or likelihood of a contrasexual puberty; and 4) Reconstruction of perineum (3). Historically, sex assignment in the newborn period was accompanied by surgical intervention to “normalize” genital appearance and function. Surgeons have traditionally favored early timing of procedures and parents may experience the desire to proceed with these surgeries for their child early in life to circumvent shame or stigma (4–7).

Despite early timing of reconstructive urogenital surgery having been the standard of care since the 1950s, intersex advocacy and human rights organizations have called for a moratorium on elective procedures until the patient is cognitively capable of being fully involved in the decision-making process. Commonly cited reasons against early surgery are the associated complications, including scarring, pain, and sexual dysfunction (8). Uncertainty over gender identity stability and loss of endogenous sex hormone production and fertility potential stemming from gonadectomy are additional reasons in favor of deferring surgery until the patient’s preferences have matured (9). Additionally, management of certain diagnoses has evolved, as is the case of complete androgen insensitivity syndrome (AIS), with some individuals preferring to retain their gonads, to allow for endogenous hormone production from puberty, and with expanding knowledge of low malignancy risk (10).

A principle of pediatric ethics states that when it is unclear what the long-term best interest of the child may be, the decision should default to a child’s right to an “open future,” and a parent is obligated to hold certain rights safe until a child is mature enough to make up their own mind (8, 11, 12). There is a caveat inherent in the open future argument as once a parent decides in either direction for a child (early surgery vs no early surgery), the child will grow and develop within the context of that decision. Given the complexity of decisions, uncertainty and lack of research with long-term outcome, and preference-sensitive options within DSD, shared decision-making is recommended.

Shared decision-making is defined as the process by which a provider and patient and/or parent reach an agreement, through collaborative deliberation, regarding the best treatment possible after discussing all options (13, 14). This model takes the patient’s and family’s values and opinions into account while relying on the expertise of the provider. Increasingly, providers are moving away from the paternalistic model of practicing medicine in DSD care as parents and adolescent or adult patients participate in the treatment planning. Providers have been strongly encouraged to practice shared decision-making since the 1990 Institute of Medicine report and many believe they are (15). However, when observed in the clinic space, many are providing what might be considered “patient-centered” without fully adhering to the principles of shared decision-making (16–18).

In the current paper, we explored how different stakeholders evaluate the decision-making process involved in making surgical decisions for patients with DSD. We aimed to understand from the stakeholders and identify themes to guide approaching this decision-making process.

## 2 Materials and methods

The methods of the first phase of the *Defining Successful Outcomes and Trade-offs* (DSOT) study, a multicenter qualitative phenomenological study, which included three collaborating sites, each with a DSD care team, are described elsewhere (19). Each of the three sites were children’s hospitals and members of the US-based Differences of Sex Development – Translational Research Network (20, 21). A summary and additional details pertaining to specific aspects of this project are provided below.

### 2.1 Participants and recruitment procedures

Participants were recruited from four stakeholder categories: 1) Individuals with a DSD (15-40 years of age) (n=24); 2) Parents (either one or both caregivers) of individuals with a DSD (child of any age under 25 years) (n= 19); 3) Healthcare providers who specialized in DSD (e.g., gynecologists, urologists) and primary care physicians (n=37); and 4) Other allied professionals with familiarity or expertise in DSD (e.g., hospital administrators, lawyers, clergy, etc.) (n=30) (**Table 1**).

**Table 1:** Participant demographics and stakeholder category.

	Individuals with a DSD (n = 24)	Parents (n = 19)	Healthcare Providers (n = 37)	Allied Professionals (n = 30)
Demographics				
Age (years): Mean (SD); range	22.1 (7.1); 15-39	37.8 (6.9); 25-52	46.1 (9.4); 28-68	49.9 (12.2); 32-78
Gender identity, n (%)				
Boy/Man	1 (4.2)	6 (31.6)	7 (18.9)	14 (46.7)
Girl/Woman	22 (91.7)	13 (68.4)	29 (78.4)	15 (50.0)
Other	1 (4.2)	0	1 (2.7)	1 (3.3)
Race/Ethnicity n (%)^a^				
African American/Black	3 (13.0)	1 (5.3)	1 (2.7)	1 (3.7)
White	16 (70.0)	17 (89.5)	30 (81.1)	24 (88.9)
Other/more than one race^c^	8 (17.4)	2 (5.3)	6 (16.2)	3 (10.7)
Hispanic	5 (20.8)	3 (15.8)	1 (2.7)	0^b^
DSD diagnosis/category^d,e^				
5α-reductase deficiency	0	1 (5.3)		
17β-hydroxysteroid dehydrogenase deficiency	0	1 (5.3)		
46,XY DSD (not otherwise specified)	2 (8.3)	0		
Ambiguous genitalia	0	2 (10.5)		
Androgen insensitivity				
Complete androgen insensitivity syndrome	4 (16.7)	0		
Partial androgen insensitivity syndrome	2 (8.3)	1 (5.3)		
Cloaca/cloacal exstrophy	3 (12.5)	4 (21.1)		
Complete gonadal dysgenesis	2 (8.3)	0		
46,XX classic congenital adrenal hyperplasia	7 (29.2)	3 (15.8)		
Hypospadias	0	3 (15.8)		
Mixed gonadal dysgenesis	2 (8.3)	0		
MRKH syndrome/Müllerian anomaly	2 (8.3)	1 (5.3)		
Ovotesticular DSD	0	2 (10.5)		
Healthcare Provider Specialties				
Endocrinology			6 (16.2)	
Genetics, Genetic Counseling, Genomics			6 (16.2)	
Pediatric & Adolescent Gynecology			4 (10.8)	
Primary Care & Adolescent Medicine			6 (16.2)	
Psychology			5 (13.5)	
Pediatric Urology & General Surgery			4 (10.8)	
Other: Child Life, Neonatology, Nursing			6 (16.2)	
Allied Professions				
Chaplaincy/Pastoral Care				7 (23.3)
Healthcare Administration				4 (13.3)
Law				2 (6.7)
Medical Ethics				4 (13.3)
Research, Clinical				5 (16.7)
Research, Social Science				4 (13.3)
Support & Advocacy Organization Leadership				4 (13.3)

a Percentages adjusted for missing data.

b n=1 participant declined to answer and n=1 missing.

c Other = Asian, Hawaii Native/Pacific Islander, or Other.

d For those recruited through clinics, diagnoses were derived from chart review; for those recruited through support or advocacy organizations, diagnoses were self-reported (n = 8).

e For parent participants, “diagnoses” reflect child’s DSD condition; SD, standard deviation; DSD, difference of sex development; MRKH, Mayer-Rokitansky-Küster-Hauser syndrome.

Eligible participants for the group of individuals with a DSD were defined as having a diagnosis or clinical presentation meeting the 2006 Consensus Statement definition for DSD (1). Individuals with a DSD were identified by clinic rosters or though patient support and advocacy organizations, and approached either in person, by phone, or in writing (i.e., letter or email). Adults with DSD were approached directly; parents were contacted for permission to contact adolescents (<18 years). All eligible participants were approached, however, only those who responded to our attempts to contact were enrolled. Exclusion criteria included lack of fluency in English, a diagnosis of Klinefelter and Turner syndromes without anatomic atypicality, and any significant reason that would prevent meaningful participation in interviews (e.g., developmental delay, current inpatient hospitalization). English-speaking parents (18 years or older) of individuals with a DSD aged 0-25 years were identified through the clinics and eligible if they had an active role in managing their child’s condition.

Providers were identified as either being a member of a DSD multidisciplinary team or a primary care physician of a patient with DSD. Allied professionals providing services to individuals who had a DSD or having familiarity with the diagnoses (e.g., hospital administrators involved with establishing best practices and care models within centers; peer support organization leaders) were targeted for recruitment. Providers and allied professionals were also identified through publications or by reaching out to advocacy leadership, through membership in DSD-related groups, or through snowball sampling. They were contacted through the collaborating site’s primary investigator *via* email or by in-person communication.

Each site’s Institutional Review Board (IRB) formally ceded oversight to the lead site’s IRB where ethical approval was granted. Consent was obtained from the parent and/or adult with DSD; assent was obtained from the teens. Participants were compensated $20 for their participation.

### 2.2 Interviews

Small group or individual interviews were scheduled and conducted by one of five moderators, all knowledgeable about DSD care, at one of three participating medical centers. An interview guide was created by study investigators and consultants, following a phenomenological model, which asked open-ended questions designed to elicit a discussion surrounding two main points: 1) What is considered a successful outcome in DSD care and 2) What are the steps for achieving these outcomes (19). Interview participants were also queried on specific topics such as outcomes at different life stages (e.g., infancy, childhood, adolescence), surgical care and outcomes, decision-making, gender identity, and fertility. Interviews were conducted either in-person or over the phone and each session was audio-recorded. The recordings were transcribed verbatim by research staff, checked for accuracy, then redacted to remove personal identifiers. Additionally, participants provided demographic variables such as age, self-identified gender, ethnicity/race, highest level of education attained and sexual preferences.

### 2.3 Data analysis

Transcribed interview files were uploaded in NVivo 12 (QSR International, Victoria, Australia) and coded at the paragraph level. Codebook themes were developed by members of the research team and validated by members not involved in codebook creation. Applying an iterative process, the codebook was updated as new themes became apparent in the data (see Suorsa-Johnson et al. for coding details) (19).

For this study, codes related to the process of decision-making and surgical outcomes specifically related to appearance and function were further queried using the keywords “surgery”, “procedure”, or “timing”. Transcript excerpts for relevant codes and text searches were reviewed by three authors (EW, MG, and KS–J) and a research assistant (CM). Authors independently reviewed themes and collaboratively organized and consolidated themes to ensure reliable analysis. Themes were reviewed by the larger research group and changes were made following group discussion. Preliminary review of transcripts revealed all participants were in favor of surgery in cases where it was necessary to preserve life or function (e.g., passing urine or stool). Data analyses are limited to surgical decision-making in non-emergent cases.

## 3 Results

Several themes emerged after reviewing all relevant codes. Different stakeholders offered varying perspectives on each topic as part of the broader discussions (see also **Table 2**).

**Table 2:** Themes.

Theme	Definition/Examples
***What is the nature of the decision?***	• Given phenotypic differences across DSD, surgical management decisions differ, e.g., those targeting appearance, function, or both, and involving genitoplasty or gonadectomy.
***Who should be involved in the surgical decision-making process?***	• Preferences for who should be involved in surgical decision-making discussions included patients, parents, and providers. • The desire to include patients posed difficulties as aspects of the decision-making process occur at a time when many are too young to be meaningfully involved. • When pediatric patients are too young to be meaningfully involved in surgical decision-making, are surgical decisions the responsibility of parents or must parents defer intervention until the child can provide meaningful assent or consent.
***When should conversations about surgery occur?***	• Discussion about the patient’s condition should precede discussion about surgery; full discussion should precede final choices about surgical management. • Timelines varied by participant, centering on “when developmentally appropriate” – estimated starting from ages between middle childhood and adolescence.
***Barriers to surgical decision-making***	• Pressure associated with anticipated stigmatization, lack of information, misinformation, culture, and religion, as well as worry about decisional regret and choosing an option for which their child would later express disagreement (regardless of the choice for or against surgery during early childhood) served as barriers.
***What are the elements in surgical decision-making?***	• Identified needs included support and information from both those within and outside the healthcare team. • Informational needs included knowledge about the condition, all available options for its clinical management, long-term effects of options, and an examination of trade-offs between options.
***What should be the approach to decisions regarding surgery?***	• Recommended approaches emphasized patient- and family-centered care in which patient and familial values, beliefs, and preferences are identified and integrated into decision-making.

### 3.1 What is the nature of the decision?

Due to the variety of anatomical and functional differences in DSD, the actual decisions needing to be made varied widely. For some, the decision was about surgery to normalize genital appearance, while for others, the decision was about removal of the gonads.

“Surgery to make her genitalia look female and function and you know do whatever she needs to do for puberty and for sex and whatever else.” – Parent of individual with DSD

In some cases, the surgical decision was regarding function rather than appearance of the genitals.

“They could do a surgery where they remove some of the inside of her cheek and put it in her lining to stretch it, and if she wants children, that [surgery is] going to have to happen.” – Parent of individual with DSD

Others expressed specific feelings on the concept of choice or decision-making.

“An important thing [to consider] is [that] not making a decision is making a decision.” – Advocacy group leader

“People would be exactly how they are. And that should be just as valid of a choice as me having surgery…” – Adult with DSD

### 3.2 Who should be involved in the surgical decision-making process?

The sentiment of most participants is that the patient should be the “decision-maker” when old enough to do so, though some did feel that parents should participate in a shared decision-making model between providers, parents, and patient. One parent referred to it as a “team” discussion between parents, patient, and providers.

“We don’t really feel like our decision to defer was our choice; we felt it was [our daughter]’s right; and we protected that right.” – Parent of individual with DSD

Some participants felt that parents should not be the ones making the decisions to perform surgery.

“I’m not a big fan of giving kids surgery just because you can’t undo it.” – Adult advocate with DSD

“I think the biggest issue that was done to me was that … I wasn’t involved in the decision.” – Adult with DSD

Others felt that parents can or should make the decision. Some parents discussed feeling decisional conflict in not wanting to make the wrong decision for their child.

“We allow parents an enormous amount of discretion to make decisions about their children and how they raise their children … which may have very profound influences on the kid and simply saying ‘no you can’t do this to your children,’ as if it’s somehow the same as child abuse, I think it’s very problematic.” – Bioethicist

“The easiest decision that I made was the surgery. Just knowing that ‘hey, she has female parts, she is going to be a female.’ That made sense to me.” – Parent of individual with DSD

“The reason why I took the decision to do that as a parent, was because first, again, I’m from a very conservative, very traditional family, our family is very focused on the family – the role of a woman, the role of a man and things like that. So that had a lot to do with it too, because I thought that [our/my daughter] should look normal.” – Parent of individual with DSD

Some individuals with DSD also felt the parents could make those decisions for young children and were happy the decision to have surgery was made for them.

“I’m not self-conscious, but if nothing were done, it would be bad.” – Adult with DSD

“So, if I were younger and [my mom] did make that decision for me, I would have been okay with it.” – Adult with DSD

### 3.3 When should conversations about surgery occur?

Participants felt that the conversations surrounding surgery and their body should occur throughout the patient’s life.

“Starting at a very young age with the simple things. You know, some parents don’t think that they are old enough … but I think, at about nine or ten, I started letting them make their own decisions at the doctors.” – Parent of an individual with DSD

Others were more specific about ages, with estimates generally falling in the teen years. Some parents felt that prior to 12 years of age, the child is still too young to understand.

“Twelve to thirteen or so. You know, we can have her sit down and talk and I’m sure by then she’ll know what she has and all that stuff. I feel like surgery would be a decision that she can make … So that way, she feels like she’s making that decision and not somebody else” – Parent of individual with DSD

“As they become developmentally mature enough, kind of turning it over to them as much as possible… 8.5 and 9 is still too young.” – Parent of individual with DSD

“So, I was old enough to make my own decision. I was fifteen or sixteen.” – Adult with DSD

If a child had surgery early in life, adolescents, and adults with DSD felt that the child should know what has happened to their body once they start to ask questions or once they can start to understand concepts about their body and what has been surgically changed.

“You know, if [a child] started to engage, I think that would be my cue as any time there is a point of engagement and questioning, that would start the conversation [about surgical procedures].” – Adult with DSD

“I mean if the kid doesn’t grasp everything that is fine and dandy. But, at the same time, I don’t think parents should ever hold any information about a child from them.” – Adult with DSD

### 3.4 Barriers to surgical decision-making

Worries about decisional regret and the potential for resentment of the child toward the parents were voiced by several parents as barriers in the decision-making process, whether the decision ended up being for or against surgery.

“One of the things that you worry about as a parent, when you have to make decisions for your kid is, ‘are they going to resent me for this’.” – Parent of individual with DSD

“Biggest worry coming out of this is that she is going to be mad at us that we didn’t do the surgery.” – Parent of individual with DSD

Parents expressed fears of their child being bullied or ostracized due to their genital difference.

“[Other parents are] worried about how their kid is going to grow up, and they’re worried about their child being bullied, or they’re worried about themselves being bullied or pushed out or ostracized.” – Parent of individual with DSD

Some participants felt physicians may be withholding information about alternative options to surgery from families or not giving them correct information.

“I’ve spoken with a couple of people who were very close to having surgery and the reasoning was that they kept having urinary tract infections and that long-term use of antibiotics was damaging which is true. What [the physicians] didn’t tell the patient was that there’s an over-the-counter medication, which sticks to the urinary tract and keeps germs from sticking to the urinary tract. So … Is it the surgery that you’re promoting or is it the health of the patient that you’re promoting? What are the other options that you’re not talking about?” – Adult advocate

Many also believed cultural or religious obligations could pressure parents to choose surgery for their child.

“Depending on the tradition, it can be this kind of sense of imposing, too, like you have to do this.” – Chaplain

“Depends on, you know, where the family is [living] and the type of lifestyle that they have … certain parts of the country … recognizing the specifics of that environment is going to play a huge part.” –Social scientist

Regardless of counsel from providers against surgical intervention, some parents felt the need to undertake surgery.

“Sometimes there are circumstances where despite the best counsel from physicians, from psychologists, from other supportive families, especially along the lines of don’t intervene, families are going to feel like they have to obtain surgical intervention typically in a cosmetic way.” – Bioethicist

“Almost all the parents that come in asking for [surgery] really want it and eventually, if we do a lot of counseling and talk to them a lot, they will come back to that is what they want- they want their kid to look as normal as possible, and they feel that that is going to make them have a better outcome … a lot of them just struggle with that idea of not doing anything.” – Gynecologist

“I think the cases that we’ve struggled with the most are ones where families from particular cultures have a very strong feeling about the desirability of having either a male child or female child. One case in particular where a newborn baby was the sixth or seventh child in the family, where all the previous children had been girls, and this is the child with the DSD. They really wanted a boy, and that was anatomically possible from a cosmetic point of view and that’s what the family wanted to do. We really wanted to be clear with them that all the risk we’ve been talking about are real and it would be better to wait for the child to make the decision itself, and this family really needed for themselves to go forward to having a boy. They ended up going through another institution for surgery.” – Bioethicist

Some parents expressed experiencing pressure from providers to do early surgery and that no one told them it was not medically necessary for their child to have surgery.

“We had doctors pushing that at us so much when [first daughter] was born. Oh my gosh, ‘testes have to come out,’ ‘she has too big of this. You gotta get this altered and this fixed.’”– Parent of individual with DSD

“It is not medically necessary to do that surgery. At all. And no one told us that for the longest time.” – Parent of individual with DSD

### 3.5 Process/Approach to decisions regarding surgery

#### 3.5.1 Elements/Information

Participants expressed several themes regarding the necessary elements for decision-making, including that the child and parents should have sufficient information to make an informed decision.

“A lot of it is really just getting to know the person; [the medical team should] spend a day with [the patient] if that is optional. And if they have the time, spend the day with them, get to know them, and basically what their family feels too because a lot of it is also family members.” – Adolescent with DSD

“Education, I think, is really important with the families about what we’re trying to achieve so they feel they can make educated decisions because sometimes there is a tradeoff.” – Urologist

To avoid bias in persuading a patient to choose one way or another, providers need to consider what is discussed, how the families are informed, and what alternatives may be offered. Participants noted that families need to understand the trade-offs and what the long-term effects of that decision would be.

“It’s a discussion about, in part, what are the values of the patient and the family themselves, you know, what’s important to them because…. surgeries carry quite the risk, quite the risk. Even apart from the actual procedure itself, it’s just the nature of anesthesia and all the rest of it. And so, helping educate the family about the specific surgical procedure and whether or not it has implications for function and sexual health down the line versus simply appearance is really important to be able to be sure families are making the choices they want to make and understand the risks, benefits for the child’s future health.” – Nurse

Participants also noted that providers should provide families with the known trade-offs associated with each treatment option, surgical and non-surgical, and take a patient-centered approach when having these ongoing discussions.

“I think it comes down to like, it’s focused around the family. And, for us as healthcare providers, we need to help them understand what these tradeoffs really mean and what the long-term effect of these might be. And I think that if we do a good job with them helping understand, then I think they’ll make, as patients, the right decisions for themselves and hopefully be happier because they have a better expectation as to what will happen.” – Pediatric surgeon

#### 3.5.2 Approach to decisions

Providers need to understand where the family is in their process and understand their backgrounds (sociocultural, religious, educational) and expectations.

“So, we see people from all walks of life and from all spiritual beliefs and all cultural differences. And it’s a very different conversation that you’re going to have with a family who has Jewish Orthodoxy as their faith base versus someone from the Middle East, who sees a woman as a wife and a child-bearer and that’s her primary role. Or a conservative Christian who has a very strong belief system about ‘we are made just as God made us and we shouldn’t be trying to change that. That we should be satisfied with the way we are made.’ So, when we speak to families, we try to be very culturally sensitive.” – Nurse

Participants felt that using educational resources or decision aid to guide decisions needing to be made could be helpful. It was also discussed in interviews how patients and parents have talked to other families and individuals with DSD and found it very beneficial in fully understanding the decision. In addition to meeting those who had opted for surgical intervention, participants also thought it would be helpful to meet with individuals who had not undergone surgery in order to garner both viewpoints. Stakeholders pointed out that there should be a team of people treating the individual with DSD, no matter the life stage, and some suggested that support groups should be a part of that team. Adolescents and adults with DSD desired open communication from providers and parents to equip them to better make informed decisions about their bodies and care, and that this communication should take place over time.

“I mean there is no set number of hours or visits [to discuss surgical options]. It’s more of a kind of a readiness gestalt in terms of how well they understand, how much they think to retain and understand what all the implications are, what the diagnosis is, or how it occurred, and what the implications are for the future. Or what the implications are with or without surgery. And that would never happen in one visit. Probably wouldn’t happen in two [visits].” – Urologist

## 4 Discussion

We explored how various DSD stakeholders within DSD care view surgery and the decision-making processes involved. This study highlights that although many feel surgery should be deferred until a patient is old enough to participate in the decision or consent for themselves, the decision is complex and there are different perspectives amongst stakeholders.

Whether the child or adolescent has the decision-making capacity due to their age or maturity level, our study showed that many participants thought that the patient should be involved in the decision. This is consistent with the literature evaluating children with chronic conditions which supports integration of the patient’s preferences into their clinical care (22). A limitation of our study, and the extant literature, is in regard to what should be done during the years when children are not yet old enough to be meaningfully involved in decision-making about their own care. There were some parents in our study who thought the decision should be theirs. However, it is unclear if this was because 1) they felt it was meant to be their decision as the proxy medical decision maker, or 2) if at the time the decision was made it was the norm to not wait until the child could be involved in the decision, or 3) that parents felt they needed to defer to providers.

Providers voiced that education and time to consider the decision and the ramifications thereof were key factors in surgical decision-making. Decision aids were also discussed as a modality to assist the families in making decisions. Provider biases and preferences should also be taken into consideration, especially in the shared decision-making framework, as their views during the counseling process may sway decisions (23).

The 2006 Consensus Statement emphasized that open communication with patients and families is essential and that their participation in the decision-making process should be encouraged (1). The 2016 Global DSD Update went a step further to recommend that the individual’s right to make decisions about their bodies or health be upheld and reiterated the need for a shared decision-making model with the individual themselves (3). The Global Update did recognize that there can be an ethical conundrum when respecting the parents’ and families’ wishes but also allowing for patient or individual autonomy: “While each of these [ethical] principles is important, striking the appropriate balance among them becomes challenging in the clinical setting. For example, respecting parents’ wishes for early genital surgery may impinge on the child’s right to participate in decision making and may reduce the child’s options for the future” (p. 176) (3). McCabe offered a framework for which an adolescent could participate in their own medical decision-making (24). There are three levels of participation: 1) Receiving information about illness status and treatment which includes procedures; 2) Shared decision-making with caregivers; and 3) Autonomous decision-making, which may include the option for the adolescent to defer to the caregiver to make decisions. The American Academy of Pediatrics (AAP) recognizes that encouraging the patient to take a bigger role in their own healthcare results in treatment compliance and confidence-building (25).

The cornerstone of shared decision-making is combining expert knowledge and equipping the patient or parent to weigh their informed preferences for option outcomes. The AAP describes the need to shift the rhetoric from parental rights toward parental responsibility by shifting the support to the child and away from the needs of the parents (25). The American Medical Association (AMA), in a special report for decision making and DSD, stated that parents are understood to be the best decision-makers, taking into account the child’s long-term interests, and in general the parents’ position should be respected. It further clarified that children should have a role in decision-making, when possible, and physicians should seek the patients’ assent to decisions about their body or care. “Physicians (and parents/guardians) should respect a child’s refusal to assent to proposed treatment. Even when immediate treatment is essential to preserve well-being, physicians should explore the child’s reason for dissent” (p.4) (26). This theme surfaced in our study with several participants stating that there needs to be a greater level of importance placed on the patient or individual with DSD, rather than the parents. Due to the nature of certain surgical interventions, which may not be medically necessary, many guidelines question the parents’ right to decide for early surgery, particularly in instances where a child is unable to participate in decision-making and where decisions may have a life-long impact, such as on sexual function (3).

There was no consensus across stakeholders on the age at which patient involvement in the decision would be appropriate, with some stating when “developmentally appropriate” but not offering a concrete age. Most adults with a DSD felt that decision-making during adolescence was appropriate. Weithorn and Campbell evaluated groups of children and adolescents, ranging from 9 to 21 years of age, who rated medical dilemmas involving care for diabetes, epilepsy, depression, and enuresis to determine whether they had the capacity to provide informed and competent medical decisions (27). Children aged 14 years and older were found to be on the same competency level as adults. Younger children who were 9 years of age seemed to lack the capacity for rationalization of treatment decision-making. This increasing competency for decision-making as one ages has been replicated in other studies assessing a child’s ability to comprehend medical or research consent/assent (28–30). This finding and sentiment aligns with the patient-parent-provider model of shared decision-making recommended by some participants in our study to include children more as they mature. A noteworthy caveat from one study was that children tend to be influenced by their parents when making treatment decisions well into their young adulthood years (31).

Even though parents were traditionally believed to be the best representatives to make such decisions, a parent’s own biases regarding stigma or shame might be driving decisions more than other factors that may be in the child’s best interests, and this should be considered in the discussions with families (5). Parents in our study verbalized a fear of their child being made fun of or ostracized later in life due to their genital difference. It is recommended that the care team discusses these fears with the parents to better understand motives for surgery and equip them to handle these concerns.

To parents, it may feel that the decision is either between early surgery or doing nothing for their child. This underscores the essential involvement and role of non-surgical providers, such as behavioral health providers. Providers in general should discuss how the decision is not surgery versus no care by including a psychosocial component, or by having a behavioral health provider as part of the team, by incorporating educational and support materials, and allowing for personalized recommendations and referrals. It is important to note that our study shares a range of thoughts and preferences of various stakeholders on multiple aspects regarding surgery and the decision-making process. These perspectives are important in highlighting a range of opinions that can be incorporated in the shared decision-making process, which should consider all the benefits and trade-offs of these complex decisions.

## 5 Conclusion

Elective surgical procedures for DSD are being justifiably scrutinized in our current society as medicine has made efforts to move away from a paternalistic approach to a patient- and family-centered model of care (15). Our study supports that shared decision-making is considered the gold standard for these difficult decisions, and stakeholders would like to see more involvement of the patient in the shared decision-making team. Developing a process for surgical decision-making in DSD by providing time to educate patients and families and weigh all options in an informed manner, as well as offering the opportunity to speak to others who have gone through the process (e.g., speaking to those who have made decisions for early surgery versus waited), while recognizing one’s personal biases, are key elements.

## Data availability statement

The original contributions presented in the study are included in the article/supplementary material. Further inquiries can be directed to the corresponding author.

## Author contributions

Study conception and design: EW, MG, KS-J, TS-K, MR, DS, KvL. Acquisition of data: EW, MG, KS-J, TS-K, MR, DS. Analysis and interpretation of data: EW, MG, KS-J. Drafting of manuscript: EW, KvL. Critical revision of manuscript: EW, MG, KS-J, TS-K, MR, DS, KvL. All authors contributed to the article and approved the submitted version.

## Footnotes

1 The term “disorders of sex development” (DSD) was proposed during the 2005 International Consensus Conference on Intersex (1). However, the word “disorder” is considered by some to be stigmatizing. As such, we use the term “differences of sex development” to recognize the controversy over labels given to these conditions. Although “Intersex” is a term preferred by some, we opted to use DSD as an expression of person-first language (2).
